# Downregulation of Epidermal Growth Factor Receptor Expression Contributes to *α*-TEA's Proapoptotic Effects in Human Ovarian Cancer Cell Lines

**DOI:** 10.1155/2010/824571

**Published:** 2010-03-04

**Authors:** Ming-Chieh Shun, Weiping Yu, Sook-Kyung Park, Bob G. Sanders, Kimberly Kline

**Affiliations:** ^1^Department of Cancer Immunology and AIDS, Dana-Farber Cancer Institute, Division of AIDS, Harvard Medical School, Boston, MA 02115, USA; ^2^School of Biological Sciences, University of Texas at Austin, Austin, TX 78712, USA; ^3^Department of Nutritional Sciences, University of Texas at Austin, Austin, TX 78712, USA

## Abstract

RRR-*α*-tocopherol derivative *α*-TEA (RRR-*α*-tocopherol ether-linked acetic acid analog) has been shown to be a potent antitumor agent both in vivo and in vitro. In this study, we investigated the effects of *α*-TEA on the expression of epidermal growth factor receptor (EGFR) family members, ErbB1, 2 and 3, and the role of ErbB 2 and 3 in *α*-TEA-induced apoptosis and suppression of Akt, FLIP and survivin in the cisplatin-sensitive (A2780S) and -resistant (A2780/CP70R) human ovarian cancer cell lines. Data show that *α*-TEA's ability to induced apoptosis was associated with reduced expression of ErbB1 (cisplatin-resistant cells), 2 and 3 (both cell types) and reduced levels of the phosphorylated (active) form of Akt; as well as, reduced levels of FLIP and survivin proteins in both cell types. Ectopic overexpression and siRNA knockdown studies showed that ErbB2, ErbB3, Akt, FLIP and survivin are involved in *α*-TEA-induce apoptosis and that *α*-TEA downregulates FLIP and survivin via suppression of pAkt, which is mediated by ErbB2 and ErB3. Thus, *α*-TEA is a potent pro-apoptotic agent for both cisplatin-sensitive and -resistant ovarian cancer cell lines in cell culture and it produces cell death, at least in part, by downregulation of members of the EGFR family.

## 1. Introduction

Ovarian cancer ranks eighth among all cancers in women in terms of estimated new cases and fifth in estimated deaths [1]. A majority of patients with ovarian cancer require treatment with cytotoxic chemotherapy. Platinum agents, cisplatin or carboplatin, are the most effective first-line treatments; however, despite initial promising responses, a high percentage of cases develop chemoresistance which significantly hinders successful treatment outcomes [2, 3]. Thus, there is a great need to develop agents for treatment of drug resistant ovarian tumors.

The epidermal growth factor receptor (EGFR) family (ErbB family) of type I receptor tyrosine kinases (RTKs) has four members: EGFR/ErbB1, ErbB2, ErbB3, and ErbB4 (also referred to as HER1, HER2, HER3 and HER4). All ErbB family members share common features including an extracellular ligand-binding domain (except ErbB2), a transmembrane domain, and an intracellular protein tyrosine kinase domain (except ErbB3). The receptors have notable differences in their sequences, which account for differential ligand-binding and diverse affinities for downstream signaling molecules [4, 5]. Data suggest that the homo- and heterodimerization between members of the ErbB family as well as the ability of their ligands to bind and activate more than one receptor help produce the complex signaling pathways of these membrane-bound proteins [6]. ErbB receptors not only play key roles in normal developmental processes but also are implicated in malignant transformation [6]. Activated ErbB receptors stimulate many intracellular signaling pathways, especially the phosphatidylinositol 3-kinase (PI3K)-AKT pathway [7]. 

Akt/protein kinase B is a family of serine-threonine protein kinases that promote cell survival and proliferation [8, 9]. Abnormal activation of Akt signaling has been reported in several human cancers [8, 10, 11] including approximately 30–40% of ovarian cancers [9, 12]. Akt promotes cell survival by mediating inactivating phosphorylation of proapoptotic proteins like Bad and caspase-9 and mediating activating phosphorylation of NF-kappaB, which controls expression of prosurvival proteins such as survivin, Bcl-2, and the caspase-8 inhibitor FLIP [13, 14]. Additionally, active Akt has been shown to confer resistance to chemotherapy in human cancers and is considered a therapeutic target [8, 11, 12]. 

Impairment of apoptotic processes can also be mediated by factors such as FLIP and survivin. FLIP and survivin are cytoplasmic proteins that function as inhibitors of caspase-8 and caspase-9/3, respectively, [15, 16]. FLIP has a similar structure to caspase-8 without the catalytic domain and thus competitively inhibits caspase-8 binding to the tumor necrosis factor family of cell surface death receptors thus blocking their apoptotic signaling [15]. Survivin is a structurally unique member in the IAP (inhibitor of apoptosis protein) family [16] and suppresses the processing and catalytic activity of execution caspases, such as casapse-9 and 3 [17]. 

Our lab has developed a vitamin E analog, 2,5,7,8-tetramethyl-2R-(4R, 8R, 12-trimethyl-tridecyl chroman-6-yloxy) acetic acid, referred to as alpha-tocopherol ether acetic acid analog (*α*-TEA), which differs in structure and function from natural vitamin E (RRR-*α*-tocopherol). *α*-TEA has an acetic acid moiety linked to the phenolic oxygen at carbon 6 of the chroman head of RRR-*α*-tocopherol by an ether linkage yielding a stable, nonhydrolyzable entity [18, 19]. Studies have demonstrated that *α*-TEA can reduce tumor burden and inhibit lung metastases when delivered by aerosol or in the diet in preclinical syngeneic transplantable mouse mammary cancer studies [18–21]; as well as in xenograft models using immune compromised mice transplanted with human ovarian, breast or prostate cancer cells [22–24]. Immunohistochemical analyses of tumor tissue from *α*-TEA treated animals indicated that *α*-TEA reduction of tumor burden was associated with increased apoptosis and reduced cell proliferation in tumor tissue [18–20, 22–24]. Cell culture studies have shown that *α*-TEA induces human ovarian, prostate and breast cancer cells to undergo DNA synthesis arrest and apoptosis, and that *α*-TEA-induced apoptosis involves activation of Fas/Fas Ligand and c-Jun NH2-terminal kinase (JNK) proapoptotic pathways; as well as, suppression of Akt, FLIP, and survivin antiapoptotic/prosurvival factors [24–27]. In this study, we investigated the effect of *α*-TEA on the expression of EGFR family proteins and studied the roles of ErbB2 and ErbB3 in *α*-TEA-induced apoptosis and suppression of Akt, FLIP and survivin antiapoptotic/prosurvival factors.

## 2. Materials and Methods

### 2.1. Chemicals

*α*-TEA (F.W. = 488.8) was prepared in house as described previously [18]. LY294002, a specific inhibitor of the p110 catalytic subunit of PI3K [28], and Wortmannin [29], a cell-permeable, irreversible inhibitor of PI3K, were purchased from Calbiochem (San Diego, CA).

### 2.2. Cell Lines and Treatments

The human ovarian A2780 cisplatin-sensitive parental cancer cell line (designated A2780S), provided by Dr. J. Rebecca Liu (University of Michigan Medical School, Ann Arbor, MI), was originally established from an untreated ovarian cancer patient [30]. The cisplatin-resistant A2780/CP70 variant (designated A2780/CP70R), provided by Dr. Michael J. Birrer (Department of Cell and Cancer Biology, National Cancer Institute, Rockville, MD), was created through intermittent exposure of A2780 cells to increasing concentrations of cisplatin (up to 70 *μ*M) in vitro [31]. A2780S and A2780/CP70R cells were grown as monolayers on plastic (Corning Plastic Ware, Corning, NY) and maintained at 37°C in RPMI 1640 (Invitrogen-Life Technologies, Inc., Carlsbad, CA), supplemented with 10% fetal bovine serum (FBS, Gemini Bio-Products, Woodland, CA), 100 IU/mL penicillin, 100 *μ*g/mL streptomycin, and 2 mM glutamine (Sigma Chemical Co., St. Louis, MO). Cultures were maintained and routinely examined to verify the absence of *Mycoplasma* contamination as described previously [32].

For experiments, the percentage of FBS was reduced to 2%. Exponentially growing A2780S and A2780/CP70R cells were plated at a density of 3 × 10^6^ in T75 flasks (10 mL) for Western analyses, or at a density of 1.5 × 10^5^/well in 12-well plates (1 mL) for apoptosis analyses. Cells were allowed to attach overnight before treatment initiation. Treatments were conducted at various concentrations of *α*-TEA in a final concentration of 0.1–0.25% ethanol. Equal volume of ethanol was used as vehicle control.

### 2.3. Cell Proliferation Assay

 To study the effects of *α*-TEA on proliferation, A2780S or A2780/CP70R cells were trypsinized, and seeded in triplicate into 96-well plates at a density of 5000 cells/well which yields a 20–30% cell density. *α*-TEA at 1.25, 2.5 or 5 *μ*M, or 2.5, 5, or 10 *μ*M were then added to A2780S or A2780/CP70R, respectively, and cells were grown in media containing 2% FBS for 1 to 3 days. Viable cell numbers were determined using Promega's CellTiter 96 Aqueous One Solution Cell Proliferation MTS assay (Promega, Madison, WI) according to the manufacturer's instructions.

### 2.4. Apoptosis Assay

Assessment of apoptosis was performed based on nuclear morphology of DAPI-stained cells as described previously [33]. Cells in which the nucleus contained clearly condensed chromatin or cells exhibiting fragmented nuclei were scored as apoptotic. Apoptotic data are reported as percentage of apoptotic cells in a given cell population sample. For each sample, a minimum of 3 counts involving a minimum of 100–200 cells/count were scored. Apoptotic data are presented as the mean ± SD for three independently performed experiments. Reagents for morphological analyses of apoptosis were purchased from Boehringer Mannheim Corp. (Indianapolis, IN).

### 2.5. Western Immunoblot Analyses

Antibodies used to detect pro- and cleaved caspase 3 (sc-7148), pro- and cleaved caspase 9 (sc-8355), HA-probe (sc-805), survivin (sc-17799), ErbB-2 (sc-284), ErbB-3 (sc-285), ErbB-4 (sc-283), and PARP (Poly(ADP-Ribose) Polymerase) (sc-7150), were purchased from Santa Cruz Biotechnology, (Santa Cruz, CA). Antibodies used to detect pro- and cleaved caspase 8 (#9746), phospho-AKT (Ser 473) (#9271), AKT (#9272), phospho-GSK-3*α*/*β* (Ser 21/9) (#9331), GSK-3*β* (#9332), and ErbB1 (#2232) were purchased from Cell Signaling Technology (Beverly, MA). Monoclonal antibody to human FLIP was purchased from Alexis Biochemical (ALX-804-428-C050); and GAPDH was made in-house. GAPDH was used for monitoring lane loads as described previously [33]. Whole cell lysates were prepared and 50 *μ*g protein was loaded per lane. Proteins were separated by using 10–15% SDS-PAGE under reducing conditions and were electroblotted onto a nitrocellulose membrane. Immunoblotting was performed using primary rabbit or mouse antibodies and peroxidase- conjugated goat antirabbit or antimouse, respectively, as the secondary antibodies (Jackson Immunoresearch Laboratory, West Grove, PA) at a 1  :  2000 dilution, followed by detection with ECL (Pierce, Rockford, IL). Quantification of band intensity was performed using Scion Image Software (Scion Corporation, Frederick, MD).

### 2.6. RNA Interference

ErbB2 and ErbB3 siRNA duplexes were synthesized by Qiagen (Valencia, CA, USA) [34]. The targeted sequences (sense strand were as follows: ErbB3: AAGAGCGACTAGACATCAAGC; ErbB2: AAGTACACGATGCGGAGACTG. Nonsilencing control siRNA (sc-37007) was from Santa Cruz Biotechnology, (Santa Cruz, CA): Cells were permitted to attach overnight and then were transiently transfected with ErbB2, ErbB3 or non-silencing control siRNAs at a final concentration of 30 nM in Lipofectamine 2000 Reagent from Invitrogen Corporation (Carlsbad, CA) following manufacturer's instructions. After one day transfection, the cells were recultured in 100 mm dish at 2 × 10^6^ cells/dish for western blot and 1.5 × 10^5^/12 well plate for apoptosis.

### 2.7. Ectopic Expression of ErbB-2 and ErbB-3

 The hemagglutinin epitope-tagged constitutively active (Myr)-AKT2 construct, HA-Myr-AKT2, and wildtype survivin expression plasmids were kindly provided by Dr. Jin Q. Cheng (Department of Pathology, Molecular Oncology, and Drug Discovery Programs, University of South Florida College of Medicine, H. Lee Moffitt Cancer Center and Research Institute, Tampa, FL) [35]. The constitutively active AKT is tagged at the carboxy-terminus with a hemagglutinin epitope tag and is modified at its aminoterminus with the c-Src-derived myristylation signal (MGSSKSKPK) [36]. The wildtype survivin expression plasmid was created by subcloning a PCR product of survivin into Myc-tagged pcDNA3.1 and confirmed by DNA sequencing analysis [37]. The wildtype His-tagged FLIP expression construct, pcDNA3.1-His-cFLIP-L, was kindly provided by Dr. John C. Reed (The Burnham Inst. La Jolla, CA) [38]. The pEGFP-C1 vector (Clontech, Mountain View, CA) was used to express enhanced green fluorescent protein (EGFP) in cells as a measure of transfection efficiency. The pcDNA3.1 expression construct containing ErbB3 was provided by Dr. Xiaofeng Li (MD Anderson Cancer Center) [39], and the expression construct of ErbB2 was kindly provided by Dr. Atanasio Pandiella (Instituto de Microbiologia Bioquimica and Centro de Investigacion del Cancer, CSIC, Universidad de Salamanca, Salamanca, Spain) [40]. A2780S and A2780/CP70R cells were plated at 1.5 × 10^6^ cells/100 mm^2^ cell culture dishes for Western immunoblot analyses and at 1.5 × 10^5^ cells/well in 12-well plates for apoptosis analyses. Cells were permitted to attach overnight and then were transiently transfected with mammalian expression vectors or appropriate vector control. Briefly, cells were washed two times with serum-free media (RPMI) and were incubated with 0.5 mL of serum-free media (OPTI-MEM I, Gibco, Grand Island, NY) containing 100 *μ*L of DNA/LipofectAMINE/Plus (Invitrogen, Carlsbad, CA) complex for apoptosis studies, and 4 mL of serum free medium (MEM-Option) containing 800 *μ*L of DNA/LipofectAMINE/Plus complex for Western immunoblot studies. DNA/LipofectAMINE/Plus reagent complex was made by first mixing 0.7 *μ*g of DNA/50 *μ*L of serum-free medium with 5 *μ*L of Plus reagent followed by 15-minute incubation, and then mixing the DNA/Plus reagent with 2 *μ*L of LipofectAMINE reagent/50 *μ*L of serum-free media followed by 14-minute incubation. After overnight transfection, cells were treated with *α*-TEA for 2 days before analyses for apoptosis or for 12 hours before Western immunoblot analyses.

### 2.8. Statistical Analysis

 All experiments were performed two or more times and experimental results were analyzed for statistical significance using 2-tail *t* test. The significance level was set at *P* < .05.

## 3. Results

### 3.1. *α*-TEA Inhibits Cell Growth and Induces Apoptosis in a Dose- and Time-Dependent Manner

**Figure 1:**
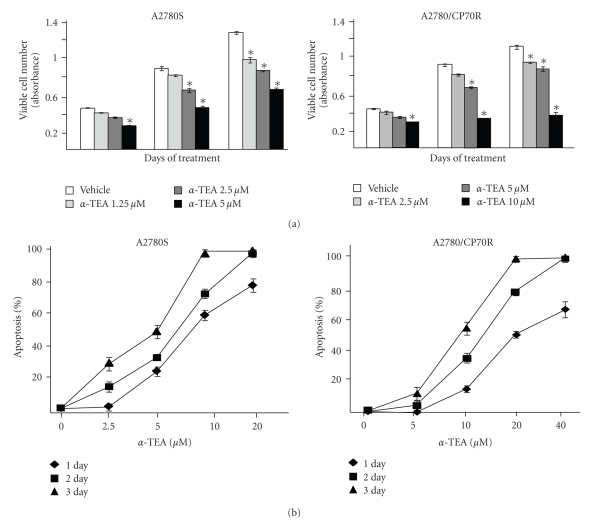
Effects of *α*-TEA on cell growth and apoptosis in A2780S and A2780/CP70R cells. (a) A2780S and A2780/CP70R cells were plated, cultured and treated as described in Section 2. Assessment of viable cell numbers (absorbance) was determined by the MTS assay following treatment with different doses of *α*-TEA for 1, 2 and 3 days. (b) Following treatment of cells with various concentrations of *α*-TEA or vehicle control for 1, 2, or 3 days, floating and adherent cells were harvested, washed, and stained with DAPI. Apoptosis was determined by counting the number of cells exhibiting condensed and fragmented nuclear morphology, and reported as percent apoptosis. Data in (a) and (b) are depicted as the mean ± SD for three independent experiments (* = *P* < .05 in comparison to vehicle control).

 Cells in monolayer cultures were treated with *α*-TEA (0, 1.25, 2.5, or 5 *μ*M for A2780S or 0, 2.5, 5, or 10 *μ*M for A2780/CP70R cells) for 1, 2, or 3 days, and viable cell numbers were determined by MTS assay. As illustrated in Figure 1(a), *α*-TEA decreased viable A2780S and A2780/CP70R cell numbers in a dose- and time-dependent manner. To confirm that the reduction in cell viability following *α*-TEA treatment was due to the induction of apoptosis, cells were treated with different levels of *α*-TEA for 1, 2, and 3 days, and apoptosis was measured by morphological analyses of cells stained with the DNA dye DAPI (Figure 1(b)). A2780S cells treated with 2.5, 5, 10, or 20 *μ*M *α*-TEA for two days exhibited dose dependent apoptosis of 16, 34, 72, and 98 % apoptotic cells; whereas, A2780/CP70R cells treated with two-fold higher levels of *α*-TEA (namely, 5, 10, 20, or 40 *μ*M) for two days exhibited dose dependent apoptosis of 5, 34, 75, and 97 % apoptotic cells (Figure 1(b)). EC50 values for 2-day *α*-TEA treatments of A2780S and A2780/CP70R cells were 5.6 and 13 *μ*M, respectively. Vehicle control treated cells of either type exhibited a low background level of apoptosis of approximately 4%. Since treatment of A2780 and A2780/CP70R with 20 and 40 *μ*M *α*-TEA, respectively, induced optimal amounts of apoptosis, these two different concentrations were used for all the following mechanistic studies. 

### 3.2. *α*-TEA Downregulates Two Prosurvival (Antiapoptotic) Factors, FLIP and Survivin in Both Cell Types and Overexpression of Either FLIP or Survivin Partially Rescues A2780S and A2780/CP70R Cells from *α*-TEA-Induced Apoptosis

**Figure 2:**
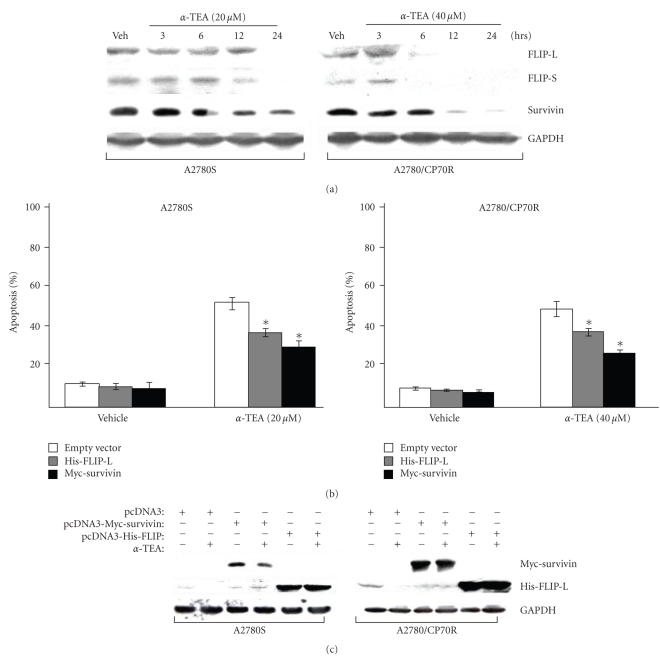
Two antiapoptotic molecules, FLIP and survivin, are downregulated by *α*-TEA in both cell types, and overexpression of either FLIP or survivin blocks *α*-TEA-induced apoptosis. (a) Cells were treated with *α*-TEA for various time periods and whole cell lysates were analyzed by western immunoblotting. ((b) and (c)) Cells were transiently transfected with empty vector control (pcDNA3), survivin (myc-survivin)- or FLIP (his-FLIP-L)-expression plasmids. After 24 hours of transfection, cells were treated with or without *α*-TEA (20 or 40 *μ*M for A2780S and A2780/CP70R, resp.) for 1 day prior to DAPI analysis for apoptosis (b), and for 12 hours prior to immunoblot analysis (c). Data in (a) and (c) are representative of a minimum of two independent experiments. Data in (b) are depicted as the mean ± SD for three independent experiments (* = *P* < .001 in comparison to vector control).

Treatment of both cell lines with *α*-TEA decreased protein levels of FLIP-L, FLIP-S and survivin in a time-dependent manner (Figure 2(a)). To assess the importance of FLIP and survivin to *α*-TEA-induced apoptosis, cells were transiently transfected with wild-type, his-tagged FLIP-L, myc-tagged survivin or empty vector (pcDNA3), and treated with either vehicle control or *α*-TEA for 24 hours. Percentage of apoptotic cells were determined (Figure 2(b)) and level of ectopically expressed proteins were measured (Figure 2(c)). Overexpression of either wild-type FLIP-L or wild-type survivin in both cell lines significantly suppressed *α*-TEA-induced apoptosis, compared to empty vector control (*P* < .001; Figure 2(b)), demonstrating that ectopic expression of FLIP-L or survivin rescues cells from *α*-TEA-induced apoptosis. Western blot analyses confirmed that transfections yielded high levels of the ectopically expressed proteins (Figure 2(c)). Please note, the Western blot depicted in Figure 2(c) is from a short exposure time that is inadequate for visualizing endogenous wildtype survivin expression. These results demonstrate that *α*-TEA downregulation of FLIP-L and survivin is required, at least in part, for maximum induction of apoptosis.

### 3.3. *α*-TEA Decreased Levels of Phosphorylated (Active) Akt

**Figure 3:**
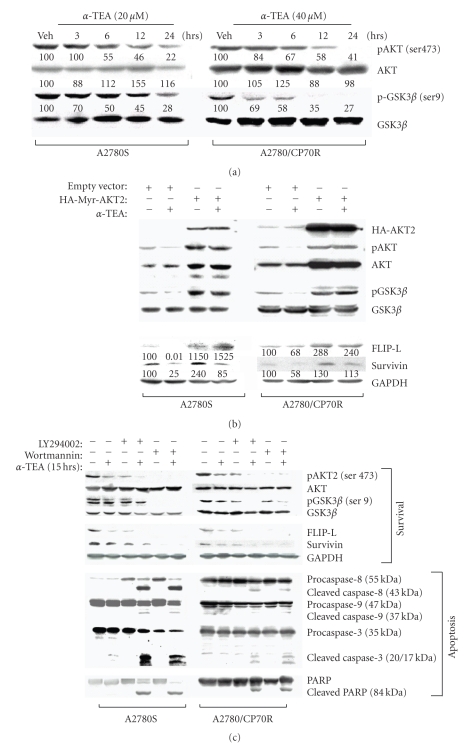
*α*-TEA decreased levels of the phosphorylated (active) form of Akt and its downstream substrate p-GSK3*β*. Overexpression of constitutively active Akt blocks *α*-TEA's ability to downregulate FLIP and survivin, while combinations of *α*-TEA and chemical inhibitors of PI3K are better at inducing apoptosis and downregulating FLIP and survivin than single treatments. (a) Cells were treated with 20 *μ*M (A2780S) or 40 *μ*M (A2780/CP70R) of *α*-TEA for 3, 6, 12, and 24 hours or vehicle control for 24 hours. Cell lysates from each treatment were analyzed by immunoblotting for pAkt, total Akt, pGSK3*β* (Ser-9), and GSK3*β*. Numbers cited under lanes represent densitometric analyses of *α*-TEA treated samples compared to vehicle control treated samples normalized for any lane load differences. (b) Immunoblotting analyses were conducted on whole cell lysates from A2780S and A2780/CP70R cells transiently transfected with HA-Myr-AKT2 plasmid, following treatment with *α*-TEA at 20 or 40 *μ*M, respectively, for 12 hours. (c) Cells were treated with two PI3K inhibitors, LY294002 (10 *μ*M) or wortmannin (1 *μ*M), and cultured with or without *α*-TEA (10 or 20 *μ*M for A2780S and A2780/CP70R cells, resp.) for 15 hours. At the end of the treatment period, cells were collected and total proteins were extracted for Western blot. All data are representative of a minimum of two independent experiments.

Phosphorylation of Akt at Ser 473 is required for its full activation [41]. *α*-TEA decreased the levels of phospho-Akt (Ser 473) while having no major effect on levels of total Akt protein expression in both ovarian cancer cell types in a time-dependent manner (Figure 3(a)). To verify that the decrease in phosphorylation status of Akt is correlated with decreased kinase activity, the phosphorylation status of GSK3*β*, a substrate of Akt, was assessed following *α*-TEA treatments. Reduced phosphorylation status of pGSK3*β* (Ser 9) was detected with little to no corresponding decreases in protein levels, indicating that *α*-TEA treatments inhibited Akt activity (Figure 3(a)).

### 3.4. Overexpression of Constitutively Active Akt Blocks *α*-TEA's Ability to Reduce FLIP and Survivin Levels

Studies were conducted to determine if *α*-TEA downregulation of FLIP and survivin is mediated by Akt. A constitutively active form of Akt2 (HA-Myr-AKT2) was overexpressed in both cell types. Cells transfected with the pcDNA3 vector alone served as control. Data show that cells transfected with the constitutively active form of Akt exhibited inhibition of *α*-TEA's ability to reduce FLIP and survivin expression (Figure 3(b)). These data suggest that Akt is an upstream mediator for both FLIP and survivin and *α*-TEA downregulation of FLIP and survivin is mediated via suppression of Akt.

### 3.5. Inhibition of Akt Using PI3K Inhibitors Enhances *α*-TEA's Ability to Induce Apoptosis and Reduce FLIP and Survivin Levels

To further investigate the function of active Akt in the regulation of FLIP and survivin, we used PI3K inhibitors LY294002 [28] and wortmannin [29] to determine whether reduced Akt activity effects *α*-TEA-induced apoptosis and FLIP and survivin protein expression. Data show that both LY294002 and wortmannin effectively inhibited the levels of phosphorylated Akt and GSK3*β* in both cell lines without markedly changing total protein levels (Figure 3(c), first to fourth panels). Likewise, FLIP and survivin expression levels were decreased by treatment with either PI3K inhibitor or *α*-TEA singly, and further decreased by co-treatments (Figure 3(c), fifth and sixth panels). Cleavage of caspases-8, -9, and -3 were enhanced by combination treatments. Likewise, PARP proteolytic cleavage as a measure of apoptosis showed that LY294002 and wortmannin augmented the apoptotic response induced by *α*-TEA (Figure 3(c), last 4 panels). 

### 3.6. *α*-TEA Downregulates ErbB Protein Levels in Both Cell Lines

**Figure 4:**
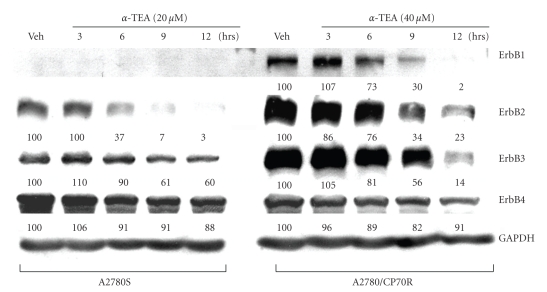
*α*-TEA downregulates ErbB1, -2, or -3 receptors in A2780S and A2780/CP70R cells. A2780S and A2780/CP70R cells were treated with 20 *μ*M and 40 *μ*M of *α*-TEA, respectively, for 3, 6, 9, and 12 hours or vehicle control for 12 hours. Cell lysates from treatment groups were analyzed by immunoblotting for ErbB1, ErbB2, ErbB3 and ErbB4 protein levels. GAPDH levels were determined to verify lane loads for densitometric analyses with vehicle control given a value of 100. Data are representative of two or more experiments.

ErbB family members play important roles in regulating epithelial cell proliferation and survival via their downstream mediators, including PI3K/Akt [7]. Therefore, we investigated whether *α*-TEA suppresses Akt/FLIP and survivin pathways via targeting ErbB family members in A2780S and A2780/CP70R cells. Western immunoblot data (Figure 4) show that A2780S cells expressed undetectable ErbB1 and lower levels of ErbB2 and ErbB3 in comparison with A2780/CP70R cells. *α*-TEA at 20 or 40 *μ*M for 3, 6, 9, or 12 hours in A2780S and A2780/CP70R, respectively, decreased ErbB1 (A2780/CP70R), and ErbB2 and ErbB3 in both cell lines (Figure 4). GAPDH levels were used to normalize densitometric values for any variation in lane loads.

### 3.7. Investigation of Causal Roles of ErbB2 and ErbB3 in Akt Signaling in *α*-TEA-Induced Apoptosis

**Figure 5:**
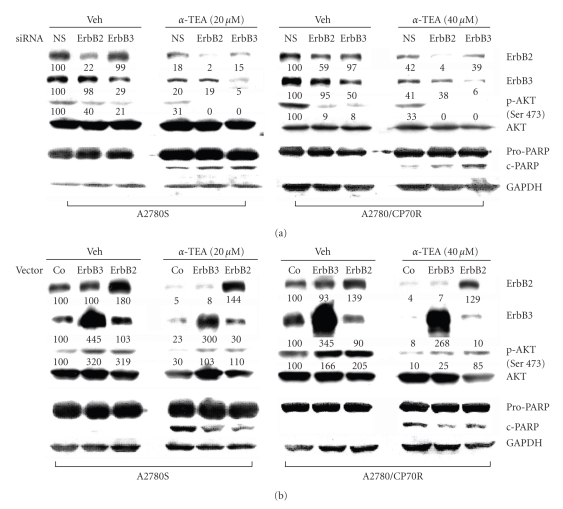
Downregulation of ErbB2 or -3 using siRNAs sensitize A2780S and A2780/CP70R cells to *α*-TEA-induced apoptosis and inhibit Akt phosphorylation; whereas overexpression of ErbB2 or -3 blocks *α*-TEA-induced apoptosis and enhances phosphorylation of Akt. (a) Cells were transfected with 20 *μ*M of sequence-specific synthetic siRNAs against ErbB2 or -3. Nonsilencing (NC) siRNA-transfected cells were used as control. Cells were treated with either vehicle or *α*-TEA for 9 hours and then lysed. 50 *μ*g of total lysate protein was used for detection of ErbB2, ErbB3, pAkt (Ser 473), total Akt, and PARP by Western blot. Data are depicted as the mean ± SD for three independent experiments. (b) Cells were transiently transfected with empty vector control (pcDNA3), ErbB2 or ErbB3 plasmids. After 24 hours of transfection, cells were treated with or without *α*-TEA for 9 hours, and whole cell extracts examined by immunoblotting. Numbers cited under lanes represent densitometric analyses for comparative purposes. Data are representative of three independent experiments.

In this study, we tested if *α*-TEA down regulation of ErbB2 or ErbB3 contributes to *α*-TEA-induced suppression of pAkt and *α*-TEA-mediated apoptosis. First, we examined the effects of functional knockdown of ErbB2 or ErbB3 using siRNA. Data show that ErbB2 and ErbB3 targeted siRNAs produced reductions in levels of pAkt in comparison to nonsilencing siRNA treated controls (Figure 5(a)). As predicted, knockdown of ErbB2 and ErbB3 enhanced *α*-TEA suppression of pAkt and increased *α*-TEA-mediated apoptosis as measured by PARP-cleavage (Figure 5(a)). 

As shown in Figure 5(b), ErbB2 and ErbB3 were successfully over expressed and *α*-TEA's ability to reduce pAkt levels and induce apoptosis were diminished but not totally eliminated, suggesting that *α*-TEA might still be an effective anticancer agent even in cases of highly elevated ErbB mediated survival. 

Collectively, these results show that ErbB2 and ErbB3 control basal, constitutively active levels of pAkt and down regulation of ErbB2 and ErbB3 contributes to *α*-TEA-induced suppression of pAkt and induction of apoptosis in both A2780S and A2780/CP70R human ovarian cancer cells. Conversely, ectopic over expression of ErbB2 or ErbB3 limited *α*-TEA's ability to induce apoptosis. 

## 4. Discussion

**Figure 6:**
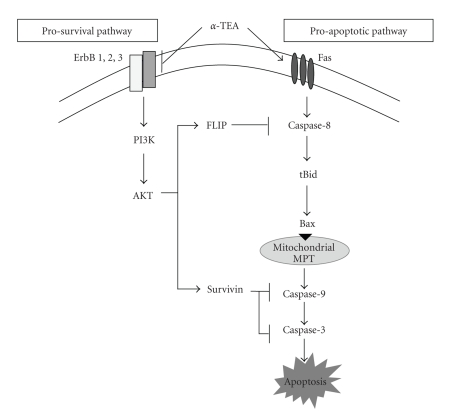
Schematic of signaling pathways involved in *α*-TEA-induced apoptosis in human ovarian cancer cell lines. Based on previously reported data [27] and data reported here, we propose both blockage of prosurvival and activation of proapoptotic signaling pathways are involved in *α*-TEA-induced apoptosis of human ovarian cancer cell lines. *α*-TEA triggers activation of the proapoptotic Fas (CD95) pathway, leading to a caspase-8- and mitochondria/caspase-9-dependent proapoptotic cascade [27]. Additionally, *α*-TEA downregulates ErbB1 (in the A2780/CP70R cells) and ErbB2 and ErbB3 in both A2780 and A2780/CP70R cell lines, leading to suppression of PI3K/Akt signaling and expression of the downstream antiapoptotic factors FLIP and survivin, which potentiates the *α*-TEA-induced proapoptotic cascade via enhancing the activation of caspase-8 and caspase-9/3, respectively, [15–17].

Data in this paper showed the following: (i) *α*-TEA is an effective stand alone anticancer agent for human ovarian cancer cell lines in that it inhibits both cisplatin-sensitive and -resistant ovarian cancer cells growth in culture by both decreasing cell proliferation and inducing apoptosis. (ii) The downregulation of ErbB2 and ErbB3/Akt/FLIP and survivin signaling events is necessary for *α*-TEA-induced apoptosis, and (iii) ErbB1 is highly expressed in the A2780/CP70R cells and below levels of detection in the A2780S cells, suggesting that ErbB1 may play a role in cisplatin resistance. Taken together with previous data that showed that *α*-TEA induces apoptosis via Fas Fas(CD95)/FasL mitochondrial dependent signaling events [27], we have summarized our current understanding of *α*-TEA induced apoptosis in human ovarian cancer cells in Figure 6. *α*-TEA is an effective anticancer agent not only because it triggers apoptosis via activation of membrane death receptor Fas (CD95)-mediated proapoptotic signaling but also because it downregulates ErbB family members and their downstream antiapoptotic effectors. 

Vitamin E analogs have not been extensively studied in ovarian cancer. Previously, we reported that *α*-TEA in combination with cisplatin significantly reduced A2780/CP70R ovarian cancer tumor burden and lung metastasis in comparison to single treatments [22], and that the apoptotic properties of *α*-TEA in human ovarian cancer cells was mediated, at least in part, via Fas (CD95) mitochondrial-dependent apoptotic signaling pathway [27]. Studies by Shanker et al. [42] showed that the succinate analog of RRR-*α*-tocopherol, vitamin E succinate, in cell culture inhibited the growth of MDAH2774 human ovarian tumor cells via extrinsic and intrinsic apoptotic pathways. In a study that compared the efficacy of *α*-TEA versus vitamin E succinate, we showed *α*-TEA was a more effective anticancer agent because esterase activity in ovarian cancer cells clipped off the succinic moiety of vitamin E succinate, yielding RRR-*α*-tocopherol which does induce apoptosis [26]. The ability of *α*-TEA to induce apoptosis in human ovarian cancer cell lines is not restricted to A2780 and A2780/CP70R cells but has been shown in a number of human ovarian cancer cell lines including 2008, 2008-C13, Hey, OVCA-429, OVCA-433, OVCA-432 and SK-OV-3 [26]. 

The ability of *α*-TEA to induce apoptosis in both A2780S and A2780/CP70 ovarian cancer cells requires both downregulation of ErbB-mediated prosurvival factors (ErbB/Akt/FLIP and survivin) and activation of Fas-mediated mitochondrial dependent apoptosis, two complementary and necessary events. 

The membrane associated epidermal growth factor receptor family (ErbB) members possess protein tyrosine kinase activity, are involved in cell survival and proliferation and are amplified in many cancers [43]. Amplification of ErbB2 protein is found in approximately one third of ovarian cancers and is an indicator of poor prognosis in advanced disease [44]. ErbB2 initiates several signaling networks involved in a variety of cellular processes, including PI3K/Akt [44]. Akt is constitutively active in ovarian cancers, and contributes to tumor cell survival by promoting the expression of survivin [37, 45]. 

Although direct measurements of Akt activity were not performed, decreased phosphorylation status of a downstream target of Akt, namely GSK3*β* was observed following *α*-TEA treatment, indicating that *α*-TEA-is downregulating Akt activity. Akt was shown to play a role in *α*-TEA induced apoptosis since expression of constitutively active Akt2 partially prevented *α*-TEA-induced apoptosis, and chemical inhibition of the PI3K/Akt pathway augmented *α*-TEA-induced apoptosis as measured by caspase-8 and casapse-9 activation, as well as PARP cleavage. Furthermore, the ability of *α*-TEA, via downregulation of Akt, to reduce FLIP and survivin provide further evidence that ErbB/Akt/FLIP/survivin signaling events help maintain ovarian cancers. 

Based on studies reported here that suppression of Akt, FLIP or survivin in the absence of *α*-TEA did not induce apoptosis, suggests that these ovarian cancer cells are not “addicted” to PI3K/Akt/cFLIP/survivin for survival. Rather data show that suppression of ErbB/Akt/FLIP/survivin antiapoptotic pathway cooperates with *α*-TEA-induced death receptor-mediated mitochondrial-dependent apoptotic cascade to sensitize the cells to cell death signals. As depicted in Figure 6, inhibition of FLIP and survivin are predicted to impact the death signaling pathway at both initiation (caspase 8) and execution (caspases 9 and 3) phases [15, 16]. Both FLIP and survivin have been implicated in contributing to cisplatin resistance in ovarian cancer [27, 46]. Therefore, downregulation of ErbB/Akt/FLIP and survivin prosurvival pathway by *α*-TEA not only enhances *α*-TEA-induced apoptosis but may also sensitize ovarian cancer cells to other proapoptotic agents. 

## 5. Conclusion

Aberrant activation of ErbB receptors and downstream PI3K/Akt signaling contributes to the development of many cancers, including ovarian cancer. This report demonstrates that *α*-TEA is a potent inducer of apoptosis in both cisplatin-sensitive and -resistant human ovarian cancer cell lines in culture. *α*-TEA's ability to initiate apoptosis is enhanced by its ability to downregulate ErbBs and subsequent downstream prosurvival mediators, Akt, and Akt mediated FLIP and survivin, yielding a dual-acting agent. A more complete understanding of *α*-TEA's multiple actions, not only adds to our basic understanding of dysregulated signaling in cancer pathophysiology but hopefully will aid in selecting the proper application of *α*-TEA in the clinic.
